# Serum Ghrelin Levels in Saudi Obese Asthmatic School-Children—Correlation with Interleukin-4, Interleukin-5, and Interleukin-21

**DOI:** 10.3390/ijerph17051656

**Published:** 2020-03-04

**Authors:** Mohammed Saeed Al-Ayed, Khaled Sadeq Al-Shaibari, Dhafer Alshehri, Mohammed Jamaan Alzahrani, Iman Nasser, Hamdan Saad Alaamri, Wed Ahmad Alaseeri, Ahmed A. Mahfouz, Saeed Ali Alsareii, Ahmed Morad Asaad, Aamir Magzoub, Mohammed Ansar Qureshi, Ehab Elagab, Elhashimi Eltayb Hassan, Mohammed Helmy Faris Shalayel

**Affiliations:** 1 Departments of Pediatrics, College of Medicine, Najran University, Najran P.O. Box 1988, Saudi Arabia; drmzayed2000@yahoo.com (M.S.A.-A.); khalid22ye@gmail.com (K.S.A.-S.); dr.zhafer@hotmail.com (D.A.); pacemaker2020@yahoo.com (M.J.A.); imansharaf18@yahoo.com (I.N.); 2 Ministry of Defense, Armed Forces Medical Services, Najran, Saudi Arabia; dr.h.38@hotmail.com; 3 Ministry of Health, Najran 11176, Saudi Arabia; wd0067@hotmail.com; 4 Department of Family and Community Medicine, College of Medicine, King Khalid University, Abha 62529, Saudi Arabia; mahfouz2005@gmail.com; 5 Department of Surgery, College of Medicine, Najran University, Najran P.O. Box 1988, Saudi Arabia; alsareii997@hotmail.com; 6 Department of Microbiology, Zagazig University, ‎Ash Sharqiyah 44519, Egypt; ahmedmoradasaad@hotmail.com; 7 Department of Physiology, College of Medicine, Najran University, Najran P.O. Box 1988, Saudi Arabia; aamirmagzoub70@hotmail.com; 8 Department of Microbiology, College of Medicine, Najran University, Najran P.O. Box 1988, Saudi Arabia; mq_ansar@yahoo.com; 9 Department of Pathology, College of Medicine, Najran University, Najran P.O. Box 1988, Saudi Arabia; ehabajab@hotmail.com; 10 Department of Clinical Biochemistry, College of Applied Medical Sciences, Najran University, Najran P.O. Box 1988, Saudi Arabia; alhashimihassan2018@gmail.com; 11 University of Hafr Al-Batin, Hafr Albatin 39524, Saudi Arabia

**Keywords:** ghrelin, interleukin-4 (IL-4), interleukin-5 (IL-5), interleukin-21 (IL-21), obesity, asthma

## Abstract

Ghrelin is a peptide hormone with direct or indirect effects on obesity and asthma. More data are required to understand the effect of ghrelin on the control and pathogenesis of these diseases. The aim of this study was to evaluate ghrelin levels in selected groups of children to identify the association between serum ghrelin, obesity, and the severity of asthma. The study included 401 school children selected from the Najran area and grouped into non-obese asthmatics, obese asthmatics, obese non-asthmatics and controls (non-obese non-asthmatics). Blood levels of ghrelin, interleukin (IL)-4, IL-5 and IL-21 were determined by ELISA. The mean ghrelin values were insignificantly increased in obese children compared with non-obese children. The highest blood ghrelin values were in the non-obese asthmatic group. Serum ghrelin, IL-4 and IL-21 levels were significantly increased in asthmatic children compared with non-asthmatic children (*p* < 0.05), and there were significant positive correlations between ghrelin and IL-4, IL-5, and IL-21 in asthmatic children. Furthermore, ghrelin, IL-4, and IL-21 levels were significantly higher in uncontrolled asthmatics compared with controlled-asthmatic children (*p* < 0.05). Asthma was the only significant risk factor for high ghrelin values. This study provides evidence supporting the anti-inflammatory role of ghrelin in the pathogenesis of asthma. Asthma might be considered as an important determinant of high ghrelin values in children.

## 1. Introduction

The World Health Organization has reported that nearly two billion people are overweight and obese [1], while 334 million people worldwide have asthma [2]. The coexistence of both diseases in one person is common, which may result in the negative distinct condition in contrast with other patients [3]. There is reported evidence that the distribution rate of obesity is significantly increased in asthma patients compared with non-asthmatic controls [4]. However, although there is a low reported prevalence of asthma in Saudi Arabia, and a recognized number of patients have uncontrolled disease [5]. A physiological link between asthma and obesity through chronic inflammatory responses has been suggested in the literature. Therefore, weight control is important to manage asthma [3].

Adipose tissue secretes mediators including adipokines and cytokines, which may harmfully influence the airways. Many reports have revealed the potential role of adiponectin (decreased in obesity) and leptin (elevated in obesity) in allergic asthma [6,7]. Bronchial asthma in obese patients differs from other forms of disease in which obese patients present with a severe attack and poor response to steroids. These findings suggest obesity is a causal factor of asthma [8,9]. Ghrelin, an endogenous ligand for the growth hormone receptor, counteracts the action of leptin to regulate energy balance [10,11]. Plasma levels of ghrelin, mostly originating from the intestines, normally increase before meals, particularly after a long fasting period [11,12,13], whereas the level decreases after carbohydrate and fat meals in rats [13]. In addition, ghrelin improves airway hyper-responsiveness and asthma symptoms in rats [14].

There is a strong link between asthma and obesity regarding serum levels of leptin and adiponectin [15]. Significant higher plasma levels of ghrelin and visfatin in asthmatic patients may imply that ghrelin exerts an anti-inflammatory effect in asthma, suggesting that it might be a new anti-inflammatory drug for asthmatic patients [16]. However, targeting the ghrelin receptor may induce unacceptable adverse effects and thus may have limited clinical use [17]. Nonetheless, in another report, there was a strong negative correlation between plasma ghrelin and serum IgE levels, in which ghrelin suppressed IgE production [18].

This study aimed to identify the correlation between serum levels of ghrelin, obesity, and asthma with the possible role of some associated inflammatory cytokines—particularly IL-4, IL-5 and IL-21—in children to investigate ghrelin as a future target in the management of asthma.

## 2. Materials and Methods

### 2.1. Study Design and Setting

This study was a cross-sectional study of a representative sample of Saudi schoolchildren in Najran, Southwestern Saudi Arabia. This work has been carried out in accordance with the Code of Ethics of the World Medical Association (Declaration of Helsinki) for experiments involving human subjects. The Ethics and Research Committee of the College of Medicine, Najran University approved the study protocol. Written informed consent was obtained from the parents or legal guardians of the students.

The sample size was calculated using the formula n = Z^2^P (1-P)/d^2^ [19], where P is the expected prevalence, estimated as 4.05% in Saudi Arabia (95% confidence interval (CI): 3.54%–4.62%) [5]. Therefore, the sample size for non-obese asthmatic children was 100, and the same sample size was chosen for obese asthmatics and obese non-asthmatics with 101 children in the control group (non-obese non-asthmatics). The grouping of children into obese and non-obese groups was based on their body mass index (BMI) percentile, because obesity is defined as a BMI greater than the 95th percentile for age and gender. In accordance with the guidelines of The Saudi Initiative for Asthma [20] and the Global Initiative for Asthma [21], asthma was diagnosed based on a history of recurrent or chronic chest symptoms such as coughing, wheezing, difficulty breathing, and chest tightness that demonstrated clinical reversibility with short-acting bronchodilator treatment. Symptom score in children with asthma was assessed according to a six-domain asthma symptom score that includes dyspnea, tightness in the chest, wheezing during the day, wheezing during the night, and daily performance [22].

Children with asthma had not received any anti-inflammatory treatment such as corticosteroids. Children with any other acute or chronic disease, including acute upper or lower respiratory tract infection, were excluded along with children who had received treatment for asthma within the previous 3 months.

### 2.2. Biochemical Evaluation

For the determination of ghrelin and interleukins-4, -5 and -21, venous blood (2 mL) was drawn at 9:00 a.m. after an overnight fast. Blood samples were left for 1 h to clot at room temperature. The serum was separated by centrifugation at 1200× *g* for 10 min and stored at −80 °C. Sera were thawed at room temperature before measurement. The serum ghrelin concentrations were measured using the ELISA method (Desacyl-Ghrelin ELISA kit, Abnova, Walnut, CA, USA). The Desacyl-Ghrelin ELISA kit was used for the in vitro quantitative assay of ghrelin peptide based on the competitive enzyme immunoassay principle. IL-4 and IL-5 were measured using Quantikine ELISA (R&D SYSTEMS, Minneapolis, MN, USA) kits, whereas IL-21 was measured using a LifeSpanBioSciences (LS Bio, Seattle, WA, USA) ELISA kit.

### 2.3. Statistical Analysis

Data were coded, validated and analysed using the SPSS version 23 software package. Frequency, percentage, arithmetic mean, median and mode were used to present the data. The Mann–Whitney test and Kruskal–Wallis tests were used as tests of significance at a 5% level of significance, while the association between variables was estimated by Pearson’s correlation. To identify potential risk factors, binary logistic multivariate analysis, adjusted odds ratio (aOR) and antecedent 95% confidence intervals (CI) were calculated. A receiver operating characteristic (ROC) curve was constructed to examine the predictive performance of bronchial asthma in identifying serum ghrelin level. The graphical plot demonstrated the performance of the cut-off points in terms of sensitivity versus specificity. The area under the curve (AUC) is a measure of the accuracy of a test or cut-off point; the AUC value lies between 0.5 and 1, where less than 0.7 denotes a poor classifier and 1 denotes an excellent classifier.

## 3. Results

The present study included 401 Saudi schoolchildren, including 345 males (86.0%) and 56 females (14.0%). Data for the ghrelin values (ng/mL) of the study sample of children ranged from 98 to 236 ng/mL with a mean of 189.18 ± 30.79 ng/mL.

The mean ghrelin values were insignificantly different in obese children (191.7 ± 29.61 ng/mL) compared to non-obese children (186.7.25 ± 31.79 ng/mL) (Mann–Whitney Z = 1.524, *p* = 0.127).

Table 1 shows the mean of ghrelin among the four studied groups of children. The four groups differed significantly from each other (Kruskal–Wallis chi-square test = 39.4, *p* = 0.001). The highest values were for non-obese asthmatics, while the lowest values were for non-obese non-asthmatics.

The serum concentrations of ghrelin, IL-4 and IL-21 were significantly increased in asthmatic children compared with non-asthmatic children (*p* < 0.05), while the serum concentrations of IL-5 were not significantly different among asthmatic children compared to non-asthmatic children (*p* = 0.124; Table 2).

This study included 200 asthmatic children. By using an asthma control questionnaire, the study revealed that 80 children (40%) were not asthma controlled (a score of less than 19), and that the rest were controlled asthmatics. Regarding the control of asthma, the results showed that the mean values of ghrelin, IL-5 and IL-21 among uncontrolled asthmatic children were significantly higher than the corresponding values among controlled-asthmatic children (*p* = 0.002; *p* = 0.001; *p* = 0.001, respectively), while the mean serum IL-4 levels were similar between controlled and uncontrolled asthmatic children (Table 3).

**Figure 1:**
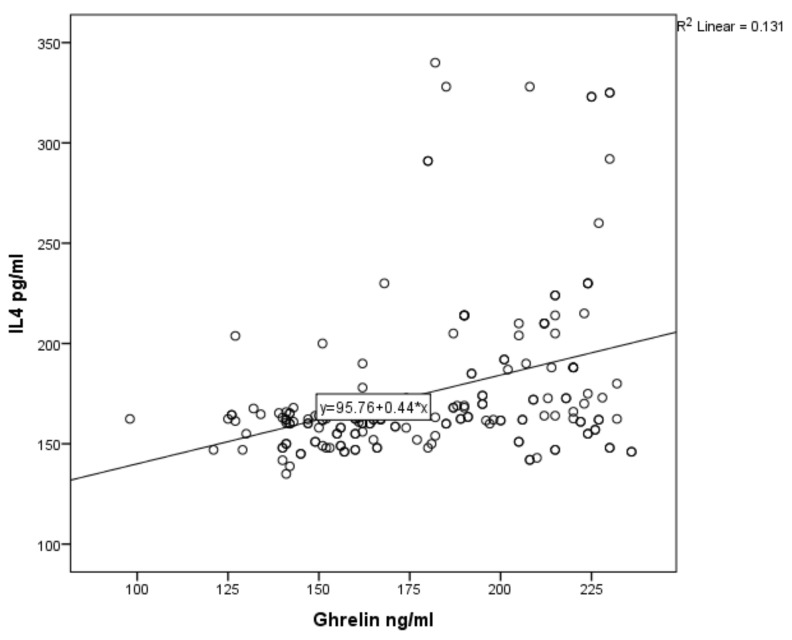
Correlation of serum ghrelin with serum IL-4 in asthmatic children.

**Figure 2:**
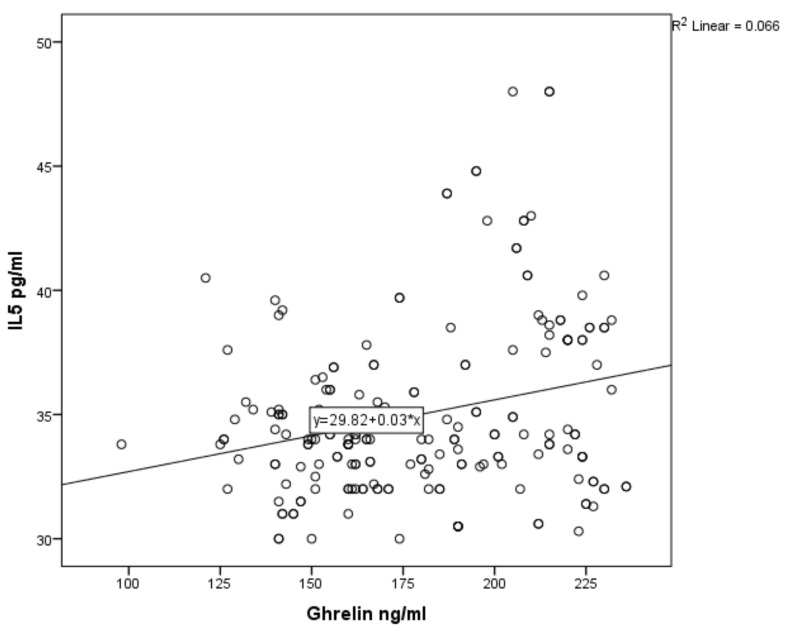
Correlation of serum ghrelin with serum IL-5 in asthmatic children.

**Figure 3:**
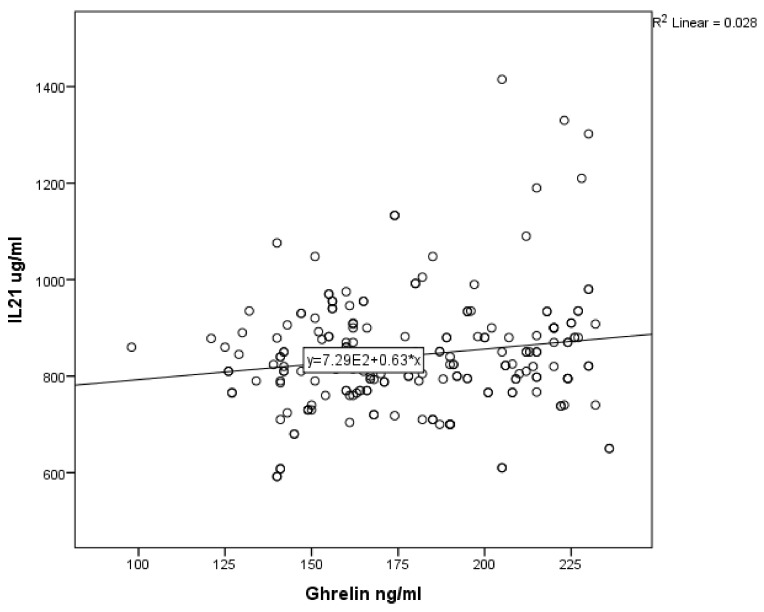
Correlation of serum ghrelin with serum IL-21 in asthmatic children.

For asthmatic children, the results of Pearson’s correlation coefficients revealed a weak positive association between serum ghrelin level and serum levels of IL-4, IL-5 and IL-21 (r = 0.362, *p* ˂ 0.001; r = 0.258, *p* ˂ 0.001; r = 0.168, *p* = 0.018; Figure 1, Figure 2 and Figure 3 respectively). In non-asthmatic children, there was no significant correlation between serum ghrelin level and the serum levels of IL-4, IL-5 and IL-21 (r = 0.161, *p* = 0.24; r = −0.037, *p* = 0.605; r = 0.086, *p* = 0.231, respectively).

The overall distribution of the ghrelin values showed that the upper quartile (75th percentile values) was 216 ng/mL. Values above or equal to 75th percentile values were regarded as high values. In multivariate logistic regression analysis, the determinants of high ghrelin values were ≥216 ng/mL (Table 4). After adjusting variables to each other, asthma was the only significant risk factor among the studied variables. Asthmatic children had more than twice the risk (aOR = 2.191, 95% CI: 1344 – 3.571) of having high ghrelin values compared with non-asthmatics.

Serum ghrelin at the optimal cut-off point of 191.5 ng/mL was poor in terms of accurately identifying asthmatic children (AUC = 0.665). Above this point, the test indicated asthma with 68.9% sensitivity and 62.3% specificity.

## 4. Discussion

Our study revealed that serum ghrelin, IL-4, and IL-21 levels were significantly increased in asthmatic children compared with non-asthmatic children, and there were significant positive correlations between ghrelin and IL-4, IL-5, and IL-21 in asthmatic children. Ghrelin is a gastric hormone with adipogenic, orexigenic, and somatotropic properties that counteracts leptin in the gastrointestinal regulation of food intake and energy balance. This interaction may have other effects in the lung, especially in asthmatics and obese patients [23,24]. In a previous study, leptin levels in obese and non-obese asthmatic children were higher than in matched non-asthmatic controls. Nevertheless, elevated leptin values in the same study were positively correlated with high levels of interferon (INF)-g (Th1 cytokine) that were attributed to inflammation around the bronchioles and the aggravation of severe asthma [22]. Ghrelin is expected to have anti-inflammatory actions that might suppress proinflammatory cytokines such as tumor necrosis factor (TNF)-α, IL-1β and IL-6, which are involved in the inflammation and pathogenesis of asthma [25,26]. There is conflicting information on ghrelin blood levels among obese or non-obese asthmatic children. For example, the role of ghrelin in weight gain in rodents was positively correlated with the administration of synthetic ghrelin, which is in accordance with our current results in humans, whereas the values of blood ghrelin were higher in obese children compared with non-obese children, although the result was not significant (*p* = 0.127). Reports from Taheri et al. and Sanchez-de-La-Torre et al. found a negative correlation between plasma ghrelin levels and BMI [26,27]. The differences in the relationship between ghrelin and body weight might be explained by specific research situations. In this study, the mean values of blood ghrelin among asthmatic children were significantly higher than among non-asthmatic children. These results are consistent with previous findings from other studies. Toru et al. and Cobanoglu et al. found that serum levels of ghrelin were significantly higher in asthmatics compared with non-asthmatic controls [16,28]. Contrary to these findings, Yuksel et al. evaluated the serum levels of leptin and ghrelin in obese and non-obese children with asthma, as well as healthy controls. The study concluded that the mean ghrelin levels in obese asthmatics were lower than in non-obese asthmatics and the control group (*p* = 0.001) [23]. Tsaroucha et al. also assessed the circulating concentrations of leptin, adiponectin and ghrelin in asthmatic patients, and they reported that these hormones have roles in the pathogenesis of asthma and its acute state [24]. Furthermore, a study of ghrelin and leptin levels to investigate immunity modulation among overweight and lean children reported that ghrelin was negatively correlated with serum IgG, IgA, and IgE, while serum IgM showed no correlation between groups [18]. In addition, ghrelin was shown to inhibit the expression of proinflammatory cytokines induced by leptin [29] Matsumoto et al. found a low level of ghrelin in asthmatics versus non-asthmatic individuals [30]. Furthermore, IgE levels were negatively correlated with ghrelin levels in obese children, suggesting that ghrelin directly or indirectly inhibits IgE synthesis. Yuksel et al. [23] reported that ghrelin levels were not correlated with symptom score or any of the disease severity parameters (r = −0.12, *p* > 0.05). However, in our study, uncontrolled asthmatics patients showed significantly higher ghrelin values compared with controlled-asthmatic children. The elevated ghrelin levels in this report might explain the competition effect of this hormone against IgE, and proinflammatory cytokines, which are concomitantly increased during asthmatic exacerbations [23]. The anti-inflammatory role of ghrelin in asthma requires more research to support the recommendations of Toru and colleagues for its use as an anti-inflammatory drug for asthmatic patients [16]. In this study, we examined the levels of IL-4, IL-5, and IL-21, which are involved in the pathology of asthma. Our results did not differ from previous studies [31,32,33], where IL-4 and IL-21 were significantly elevated in asthmatic children compared with non-asthmatic children, while IL-5 showed an insignificant increase in asthmatic children. IL-4 was associated with IgE production and allergy [34], and we found that the level of IL-4 increased among uncontrolled asthmatics in contrast to controlled asthmatic patients; however, this was statistically insignificant. Moreover, IL-21 and IL-5 levels were significantly higher in uncontrolled versus controlled asthmatic patients. IL-21 has an anti-allergic effect, evidenced by its role in the remission of allergic rhinitis in mice [35]. In addition, IL-21 has a regulatory function in humoral and adaptive immunity, where it antagonizes allergic IgE antibodies [36]. This might explain the occurrence of high levels of IL-21 among uncontrolled asthmatic patients in contrast to non-asthmatics. The concomitant increases of ghrelin and IL-4, IL-5, and IL-21 in uncontrolled asthmatic patients versus the controlled asthmatic group in this study is interesting. IL-4 and IL-5 play a crucial mechanistic role in asthma. They enhance eosinophilic and allergic inflammation, which further results in alterations in the smooth muscle cells, leading to the hyper-secretion of mucus, increased smooth muscle contractility, and airway hyperresponsiveness [37].

Therefore, we applied further statistical measures to understand this relationship. A positive correlation between ghrelin level and IL-4, IL-5, and IL-21 was determined, but there was no correlation with non-asthmatic patients.

This study suggests that this metabolic hormone might be linked with cytokines in the pathophysiology of asthma, in a similar manner to its positive anti-inflammatory effect on spinal cord injury [38] and sepsis [39].

In this study, multiple logistic regression analysis was used to identify the determinants of ghrelin levels above the 75th percentile value of 216 ng/mL after adjusting variables to each other. We found that only asthma was a significant risk factor. Asthmatic children have over twice the risk (aOR = 2.19, 95% CI: 1.34–3.57) of having high ghrelin levels compared with non-asthmatics. However, in contrast to our findings, previous clinical studies found that ghrelin had immunomodulatory activities through the dose- and time-dependent inhibition of IL-1β, IL-6, and TNF-α. Furthermore, ghrelin, in some studies, reduced the levels of proinflammatory adipokines and cytokines from mononuclear and T cells induced by leptin [23,27,28]. The downregulation of these cytokines might be explained by the energy imbalance induced by ghrelin, which interferes with the function of sensitive innate and adaptive immune cells [28].

Ghrelin was significantly higher among non-obese asthmatics compared to non-obese non-asthmatics, suggesting the impact of asthma on raising the ghrelin level. Similarly, ghrelin was significantly higher among obese non-asthmatics compared to non-obese non-asthmatics, suggesting the impact of obesity in raising the level of this hormone. On the other hand, ghrelin was significantly higher among obese asthmatics compared to non-obese asthmatics, suggesting the additive impact of being both obese and asthmatic. Moreover, and interestingly, there are concomitant increases of ghrelin, IL-4, IL-5, and IL-21 in uncontrolled asthmatic patients versus the controlled asthmatic group, with a direct correlation between ghrelin and these cytokines.

These results suggest the possible protective role of ghrelin on pulmonary airways. It seems that whenever there is a risk or a challenge of increases in airway resistance, such as asthma or obesity, ghrelin levels tend to increase as a compensatory protective mechanism. Therefore, a question regarding the nature of the airway protective mechanism of ghrelin arises: does this occur because of an anti-inflammatory effect? If so, the increase in the serum level of ghrelin should be accompanied by a decrease in IL-4 and IL-5, which was not the case in this study. Therefore, we thought of another possible mechanism; i.e., that ghrelin might have a direct protective effect on airway smooth muscles rather than an anti-inflammatory effect. We conducted an in vitro study on the direct relaxant effect of ghrelin on guinea pig airway smooth muscles on both intact trachea and sensitized one to mimic the asthma model. In this published study, ghrelin has shown a promising relaxing effect on carbachol-contracted tracheal smooth muscles. The effect was more evident in the intact non-sensitized than in sensitized groups (*p* < 0.05). The effect is suggested to occur partially through an epithelium-dependent mechanism because preincubation with nitric oxide (NO) and prostaglandin E2 (PGE2) inhibitors significantly reduced the ghrelin-induced relaxation. Nevertheless, the relaxing effect was not completely abolished, suggesting an epithelium-independent mechanism [40]. In the case of reactive airway disease, the airway inflammation might decrease the release of the epithelium-derived factors nitric oxide (NO) and prostaglandin E2 (PGE2) and therefore weaken the direct protective effect of ghrelin. However, ghrelin might act through an epithelium-independent mechanism, which justifies the higher ghrelin levels observed in asthmatic groups. Still, a question needs to be answered: what is the signal for the rise in ghrelin levels in asthma and obesity? Further studies are required to address this.

To understand the effect of ghrelin and cytokines in asthmatic patients, a prospective study that involves the investigation of additional inflammatory mediators and the selection of an appropriate measurement time based on the condition of patients related to a state of hunger or satiety is required. In addition, we must keep the patient’s medical status in mind as cytokines increase and decrease in acute cases. A limitation of this study was that ghrelin levels vary during the day in response to hunger and body energy, which may give misleading results. Furthermore, samples were taken from asthmatic patients at various disease stages and different unknown sub-types of asthma, leading to different cytokine expressions. In addition, ghrelin levels were obtained from serum without inclusion of protease inhibitors and this could be a limitation of our study.

## 5. Conclusions

This work revealed that serum ghrelin, IL-4, and IL-21 levels were significantly higher among asthmatic children. Interestingly, blood ghrelin, IL-5, and IL-21 levels were significantly higher among uncontrolled asthmatics compared to controlled-asthmatic children. This study also provides further evidence supporting the anti-inflammatory role of ghrelin in the pathogenesis of asthma. In this context, the elevation of either ghrelin and interleukins in asthmatics may highlight the balanced interaction between the anti-inflammatory role of ghrelin and the pro-inflammatory role of some interleukins during uncontrolled asthma. The elevation of both ghrelin and some interleukin levels in asthmatic patients may suggest that ghrelin might act through an epithelium-independent mechanism. Asthma might be considered as an important determinant of high ghrelin values in children.

## Figures and Tables

**Table 1:** Ghrelin level among different groups of children.

Variable	Number	Median	Mean	SD
Non-obese non-asthmatic	101	166	174.02	31.23
Non-obese asthmatic	100	212	199.25	27.13
Obese non-asthmatic	100	190	186.21	30.99
Obese asthmatic	100	210	197.43	27.09

**Table 2:** Ghrelin and interleukin levels among the study sample with the presence of bronchial asthma. IL: interleukin.

Parameter (Median, Mean ± SD)	Asthmatics: N (200)	Non-Asthmatics: N (201)	*p* Value
Ghrelin, ng/mL	210, 198.40 ± 27.06	178, 180.10 ± 31.63	0.001 *
IL-4, pg/mL	172.5, 191.02 ± 45.98	163, 175.51 ± 38.52	0.001 *
IL-5, pg/mL	35, 36.51 ± 6.14	34, 35.08 ± 3.58	0.124
IL-21, µg/mL	880, 896.20 ± 157.66	824, 843.45 ± 119.16	0.003 *

*: *p* < 0.05.

**Table 3:** Ghrelin and interleukin levels among the study sample by the control of bronchial asthma.

Parameter (Mean ±SD)	Controlled: N (120)	Uncontrolled: N (80)	*p* Value
Ghrelin, ng/mL	193.81 ± 27.51	204.95 ± 25.12	0.002 *
IL-4, pg/mL	190.00 ± 48.45	191.10 ± 44.46	0.214
IL-5, pg/mL	35.51 ± 5.47	38.02 ± 6.77	0.001 *
IL-21, µg/mL	857.51 ± 133.10	954.25 ± 173.70	0.001 *

*: *p* < 0.05.

**Table 4:** Multivariate logistic regression analysis of factors determining high ghrelin values.

Variables	aOR	95% CI	*p* Value
Sex: males vs. females	0.449	0.179–1.129	0.089
Obesity: obese vs. non-obese	1.057	0.669–1.669	0.812
Asthmatic: Asthmatics vs. non-asthmatics *	2.191	1.344–3.571	0.002

*: *p* < 0.05. aOR; adjusted odds ratio: CI; confidence interval.

## References

[1] Mohanan S., Tapp H., McWilliams A., Dulin M. (2014). Obesity and asthma: Pathophysiology and implications for diagnosis and management in primary care. Exp. Boil. Med..

[2] Masoli M., Fabian D., Holt S., Beasley R. (2004). Global Initiative for Asthma (GINA) program: The global burden of asthma: Executive summary of the GINA Dissemination Committee report. Allergy.

[3] Duran-Tauleria E., Rona R.J. (1999). Geographical and socioeconomic variation in the prevalence of asthma symptoms in English and Scottish children. Thorax.

[4] Su X., Ren Y., Li M., Zhao X., Kong L., Kang J. (2016). Prevalence of comorbidities in asthma and non-asthma patients. Medicine (Baltimore).

[5] Moradi-Lakeh M., El Bcheraoui C., Daoud F., Tuffaha M., Kravitz H., Al Saeedi M., Basulaiman M., Memish Z.A., Al Mazroa M.A., Al Rabeeah A.A. (2015). Prevalence of asthma in Saudi adults: Findings from a national household survey, 2013. BMC Pulm. Med..

[6] Sideleva O., Suratt B.T., Black K.E., Tharp W.G., Pratley R.E., Forgione P., Dienz O., Irvin C., Dixon A. (2012). Obesity and asthma: An inflammatory disease of adipose tissue not the airway. Am. J. Respir. Crit. Care Med..

[7] Hancox R.J., Milne B.J., Poulton R., Taylor D.R., Greene J.M., McLachlan C.R., Cowan J.O., Flannery E.M., Herbison G.P., Sears M.R. (2005). Sex Differences in the Relation between Body Mass Index and Asthma and Atopy in a Birth Cohort. Am. J. Respir. Crit. Care Med..

[8] Umetsu D.T. (2017). Mechanisms by which obesity impacts upon asthma. Thorax.

[9] Sin D.D., Jones R.L., Man S.F.P. (2002). Obesity is a risk factor for dyspnea but not for airflow obstruction. Arch. Intern. Med..

[10] Taylor B., Mannino D., Brown C., Crocker D., Twum-Baah N., Holguin F. (2008). Body mass index and asthma severity in the National Asthma Survey. Thorax.

[11] Kojima M., Hosoda H., Date Y. (1999). Ghrelin is a growth-hormone- releasing acylated peptide from stomach. Nature.

[12] Tolle V., Bassant M.H., Zizzari P., Poindessous-Jazat F., Tomasetto C., Epelbaum J., Pajot M. (2002). Ultradian rhythmicity of ghrelin secretion in relation with gh, feeding behavior, and sleep-wake patterns in rats. Endocrinology.

[13] Nakahara K., Okame R., Katayama T., Miyazato M., Kangawa K., Murakami N. (2010). Nutritional and environmental factors affecting plasma ghrelin and leptin levels in rats. J. Endocrinol..

[14] Fu T., Wang L., Zeng Q., Zhang Y., Sheng B., Han L. (2018). Ghrelin ameliorates asthma by inhibiting endoplasmic reticulum stress. Am. J. Med. Sci..

[15] Salah A., Ragab M., Masnsour W., Taher M. (2015). Leptin and adiponectin are valuable serum markers explaining obesity/bronchial asthma interrelationship. Egypt. J. Chest Dis. Tuberc..

[16] Toru Ü., Ayada C., Genç O., Şahin S., Arık Ö., Acat M., Bulut İ., Çetinkaya E. (2015). Visfatin and ghrelin: Can they be forthcoming biomarkers or new drug targets for asthma?. Int. J. Clin. Exp. Med..

[17] Mende F., Hundahl C., Plouffe B., Skov L.J., Sivertsen B., Madsen A.N., Lückmann M., Diep T.A., Offermanns S., Frimurer T.M. (2018). Translating biased signaling in the ghrelin receptor system into differential in vivo functions. Proc. Natl. Acad. Sci. USA.

[18] Okamatsu Y., Matsuda K., Hiramoto I., Tani H., Kimura K., Yada Y., Kakuma T., Higuchi S., Kojima M., Matsuishi T. (2009). Ghrelin and leptin modulate immunity and liver function in overweight children. Pediatr. Int..

[19] Suresh K., Chandrashekara S. (2012). Sample size estimation and power analysis for clinical research studies. J. Hum. Reprod. Sci..

[20] Al-Moamary M.S., Alhaider S.A., Idrees M.M., Al Ghobain M.O., Zeitouni M.O., Al-Harbi A.S., Yousef A.A., Al-Matar H., Alorainy H.S., Al-Hajjaj M.S. (2016). The Saudi Initiative for Asthma—2016 update: Guidelines for the diagnosis and management of asthma in adultsand children. Ann. Thorac. Med..

[21] Alsahn B., Alshamrani A., Alzahrani A., Alsahmi O., Alqudhybi A. (2017). Asthma Control Assessment Using Asthma Control Test among Pediatric Patients Attending a Tertiary Care Hospital in Saudi Arabia. Egypt. J. Hosp. Med..

[22] Youssef D.M., Elbehidy R.M., Shokry D.M., Elbehidy E.M. (2013). The influence of leptin on Th1/Th2 balance in obese children with asthma. J. Bras. Pneumol..

[23] Yüksel H., Sogut A., Yilmaz O., Onur E., Dinç G. (2011). Role of Adipokines and Hormones of Obesity in Childhood Asthma. Allergy Asthma Immunol. Res..

[24] Tsaroucha A., Daniil Z., Malli F., Georgoulias P., Minas M., Kostikas K., Bargiota A., Zintzaras E., Gourgoulianis K.I. (2012). Leptin, Adiponectin, and Ghrelin Levels in Female Patients with Asthma during Stable and Exacerbation Periods. J. Asthma.

[25] Müller T.D., Nogueiras R., Andermann M.L., Andrews Z.B., Anker S.D., Argente J., Batterham R.L., Benoit S.C., Bowers C.Y., Broglio F. (2015). Ghrelin. Mol. Metab..

[26] Taheri S., Lin L., Austin D., Young T., Mignot E. (2004). Short sleep duration is associated with reduced leptin, elevated ghrelin, and increased body mass index. PLoS Med..

[27] Sánchez-De-La-Torre M., Mediano O., Barceló A., Pierola J., De La Peña M., Esquinas C., Miro A., Durán-Cantolla J., Agusti A.G., Capote F. (2011). The influence of obesity and obstructive sleep apnea on metabolic hormones. Sleep Breath..

[28] Cobanoglu N., Galip N., Dalkan C., Bahceciler N.N. (2013). Leptin, ghrelin and calprotectin: Inflammatory markers in childhood asthma. Multidiscip. Respir. Med..

[29] Dixit V.D., Taub D.D. (2005). Ghrelin and immunity: A young player in an old field. Exp. Gerontol..

[30] Matsumoto Y., Toyomasu K., Uchimura N., Ishitake T. (2013). Low-molecular-weight adiponectin is more closely associated with episodes of asthma than high-molecular-weight adiponectin. Endocr. J..

[31] Moser R., Fehr J., Bruijnzeel P.L. (1992). IL-4 controls the selective endothelium-driven transmigration of eosinophils from allergic individuals. J. Immunol..

[32] Dubucquoi B.S., Desreumaux P., Janin S.A., Klein O., Goldman M., Tavernier I.I.J., Capron A., Capron M. (1994). Interleukin 5 synthesis by eosinophils: Association with granules and immunoglobulin-dependent secretion. J. Exp. Med..

[33] Gong F., Su Q., Pan Y.H., Huang X., Shen W.H. (2013). The emerging role of interleukin-21 in allergic diseases (Review). Biomed. Rep..

[34] Parronchi P., De Carli M., Manetti R., Simonelli C., Piccinni M.-P., Macchia D., Maggi E., Del Prete G., Ricci M., Romagnani S. (1992). Aberrant interleukin (IL)-4 and IL-5 productionin vitro by CD4+ helper T cells from atopic subjects. Eur. J. Immunol..

[35] Hiromura Y., Kishida T., Nakano H., Hama T., Imanishi J., Hisa Y., Mazda O. (2007). IL-21 administration into the nostril alleviates murine allergic rhinitis. J. Immunol..

[36] Sivakumar P.V., Foster D.C., Clegg C.H. (2004). Interleukin-21 is a T-helper cytokine that regulates humoral immunity and cell-mediated anti-tumour responses. Immunol..

[37] Miethe S., Guarino M., Alhamdan F., Simon H.-U., Renz H., Dufour J.-F., Potaczek D.P., Garn H. (2018). The effects of obesity on asthma: Immunometabolic links. Pol. Arch. Intern. Med..

[38] Ersahin M., Toklu H.Z., Erzik C., Akakin D., Tetik S., Şener G., Yeğen B.Ç. (2011). Ghrelin alleviates spinal cord injury in rats via its anti-inflammatory effects. Turk. Neurosurg..

[39] Wu R.-Q., Dong W., Cui X., Zhou M., Simms H.H., Ravikumar T.S., Wang P. (2007). Ghrelin Down-regulates Proinflammatory Cytokines in Sepsis Through Activation of the Vagus Nerve. Ann. Surg..

[40] Al-Ayed M.S.Z. (2018). Relaxant effect of ghrelin on guinea pig isolated tracheal smooth muscle: Role of epithelial NO and PGE2. Pflügers Archiv. Eur. J. Physiol..

